# Ingestion of Indigestible Cacao Proteins Promotes Defecation and Alters the Intestinal Microbiota in Mice

**DOI:** 10.1093/cdn/nzac129

**Published:** 2022-08-06

**Authors:** Jinichiro Koga, Kota Ojiro, Ayumi Yanagida, Takahisa Suto, Hideaki Hiki, Yuki Inoue, Chihiro Sakai, Kohei Nakamoto, Yuta Fujisawa, Ayaka Orihara, Haruka Murakami, Shintaro Hirasawa, Kengo Nakajima, Tomoko Sakazawa, Hisakazu Yamane

**Affiliations:** Department of Biosciences, School of Science and Engineering, Teikyo University, Utsunomiya, Tochigi, Japan; Department of Biosciences, School of Science and Engineering, Teikyo University, Utsunomiya, Tochigi, Japan; Department of Biosciences, School of Science and Engineering, Teikyo University, Utsunomiya, Tochigi, Japan; Department of Biosciences, School of Science and Engineering, Teikyo University, Utsunomiya, Tochigi, Japan; Department of Biosciences, School of Science and Engineering, Teikyo University, Utsunomiya, Tochigi, Japan; Department of Biosciences, School of Science and Engineering, Teikyo University, Utsunomiya, Tochigi, Japan; Department of Biosciences, School of Science and Engineering, Teikyo University, Utsunomiya, Tochigi, Japan; Department of Biosciences, School of Science and Engineering, Teikyo University, Utsunomiya, Tochigi, Japan; Department of Biosciences, School of Science and Engineering, Teikyo University, Utsunomiya, Tochigi, Japan; Department of Biosciences, School of Science and Engineering, Teikyo University, Utsunomiya, Tochigi, Japan; Department of Biosciences, School of Science and Engineering, Teikyo University, Utsunomiya, Tochigi, Japan; Department of Biosciences, School of Science and Engineering, Teikyo University, Utsunomiya, Tochigi, Japan; Department of Biosciences, School of Science and Engineering, Teikyo University, Utsunomiya, Tochigi, Japan; Department of Biosciences, School of Science and Engineering, Teikyo University, Utsunomiya, Tochigi, Japan; Department of Biosciences, School of Science and Engineering, Teikyo University, Utsunomiya, Tochigi, Japan

**Keywords:** cacao proteins, *Theobroma cacao* L., defecation, intestinal microbiota, chocolate, prebiotics, *Lactococcus*, *Oscillospira*, *Mucispirillum*, *Anaerotruncus*

## Abstract

**Background:**

In animals, the health effects of ingested cacao proteins are unknown because the proteins are difficult to extract and purify from cacao beans.

**Objectives:**

This study aimed to develop an extraction and purification method for cacao proteins and reveal the effect of ingestion of cacao proteins on defecation and intestinal microbiota in mice.

**Methods:**

Three groups of mice were fed a control diet (AIN-93 G), a cacao lignin diet (AIN-93 G containing 1.22% cacao lignin), or a cacao protein and lignin diet (AIN-93 G containing 1.97% cacao proteins and 1.22% cacao lignin) by pair-feeding for 8 d. Feces were collected as 2 bulked samples from days 1 to 4 and days 5 to 8 on each diet. The collected feces were weighed and the intestinal microbiota was analyzed by next-generation sequencing-based 16S rRNA.

**Results:**

A new extraction and purification method for cacao proteins has been developed, then found that the proteins are resistant to digestive enzymes. However, the cacao protein powder made by this method contained 34.9% of lignin in addition to 56.4% of proteins. Therefore, to reveal the effect by cacao proteins alone, the fecal weight and intestinal microbiota of mice fed the cacao protein and lignin diet were compared with those of mice fed the cacao lignin diet. The fecal weight of mice fed the cacao protein and lignin diet was significantly greater than of mice fed the cacao lignin diet. The relative abundance of *Lactococcus* and *Mucispirillum* species in mice fed the cacao protein and lignin diet was significantly higher than in mice fed the cacao lignin diet, but the relative abundance of *Anaerotruncus*, *Oscillospira*, and *Roseburia* species in mice fed the cacao protein and lignin diet was significantly lower than in mice fed the cacao lignin diet.

**Conclusions:**

Ingestion of indigestible cacao proteins promoted defecation and altered the intestinal microbiota such as *Lactococcus*, *Mucispirillum*, *Anaerotruncus*, *Oscillospira*, and *Roseburia* species in mice.

## Introduction

Constipation, one of the most common health problems worldwide, is clearly increasing due to changes in eating habits and psychological and social factors, seriously affecting health and quality of life (1). Irritant drugs that promote defecation are known to have side effects (2). A clinical study has shown that the supplementation with *Bifidobacterium infantis* alleviates the bowel movement difficulty as well as symptoms in inflammatory bowel disease (IBD) (3), suggesting that it might alleviate constipation (4). Hence, supplementation with such beneficial bacteria to improve the balance of intestinal microbiota known as “probiotics” has become a new method to alleviate constipation (5, 6).

Functional food components known as prebiotics have also attracted attention as ingredients to improve intestinal microbiota by stimulating the growth of beneficial bacteria in the colon (7, 8). Because prebiotics are not degraded by digestive enzymes, they pass into the colon and are consequently metabolized by intestinal bacteria. Among the prebiotics, oligosaccharides such as fructo-oligosaccharides and galacto-oligosaccharides are metabolized by intestinal bacteria such as *Bifidobacteria* and *Lactobacillus* and their metabolism leads to an increase in the number of these bacteria in the colon (9–16). *Bifidobacteria* produce lactic acid and acetic acid that decrease the pH in the colon. This lower pH is considered to stimulate peristalsis and decrease the colonic transit time, thus helping to alleviate constipation (17).

Chocolate and cocoa are produced from fermented, roasted cacao beans (*Theobroma cacao* L.) and are known to contain high amounts of polyphenols that have several benefits for human health, such as prevention of cardiovascular and inflammatory diseases, metabolic disorders, and cancer (18–25). However, the effects of cacao proteins on human health are still unknown, because the proteins are difficult to extract and purify from cacao beans. In the present study, we developed a new extraction and purification method for cacao proteins, then found that the proteins are resistant to digestive enzymes. Therefore, we speculated that cacao proteins in a diet would pass undigested into the colon, where they would be metabolized by intestinal bacteria to promote defecation in the same manner as oligosaccharides such as fructo-oligosaccharides and galacto-oligosaccharides. Based on this background, we investigated whether the ingestion of cacao proteins promotes defecation and alters the intestinal microbiota in mice.

## Methods

### Search for a suitable extraction and purification method of proteins from cacao beans

Cacao beans were defatted by compressing and crushed into fine powder. The defatted cacao powder (BNT12) was provided by Meiji Co., Ltd. (Tokyo, Japan).

A neutral aqueous solution was used to compare the amount of total proteins extracted from almonds, macadamia nuts, soybeans, broad beans, or cacao beans; 5 g of seeds was homogenized with 200 mL of 40 mM potassium phosphate solution (pH 6.8). Each homogenate was then agitated for 30 min at 50°C, then centrifuged at 20,400 × *g* for 20 min at 25°C. The protein concentration of the supernatant was measured by the Bradford method (Protein Assay Kit; Bio-Rad) with bovine serum albumin (BSA) as the standard (26).

For determining the most effective method to extract proteins from cacao beans, 5 g of cacao beans was homogenized in various solution: 200 mL of 40 mM sodium acetate solution (pH 3.5), potassium phosphate solution (pH 6.8), potassium phosphate solution (pH 6.8) plus 0.2% Triton X-100, or sodium carbonate solution (pH 10.0). Each homogenate was agitated for 30 min at 50°C, then centrifuged at 20,400 × *g* for 20 min at 25°C, and the protein concentration of the supernatant was measured by the Bradford method.

For determining the optimal conditions for alkaline extraction and acid precipitation of the proteins from cacao beans, the defatted cacao powder was washed twice with 50% ethanol (EtOH) at 50°C to remove polyphenols and theobromine, then the residue was washed twice with water (pH 6.0) at 50°C to remove oils and fats as described in the next section. The consequent residue was used in a series of alkaline extraction or acid precipitation tests. First, to find the best alkaline extraction condition, 30 g of the residue added to 500 mL of various sodium hydroxide (NaOH) solutions (final pH 10.0–12.0) was agitated for 60 min at 50°C. The amount of proteins extracted was measured by the Bradford method. Next, for finding the most effective acid precipitation method of cacao proteins, the protein extract obtained in the NaOH solution (pH 11.5), which had the highest protein yield, was precipitated with hydrochloric acid (HCl) solution (final pH 3.0–5.0). The amount of protein precipitated was measured by the Bradford method.

In the search for a method showing the highest protein yield, each cacao protein powder was extracted and purified from the defatted cacao powder under various alkaline extraction conditions (pH 10.5–12.0) according to the method described in the next section. The amount of nitrogen in each cacao protein powder and defatted cacao powder was measured by the Kjeldahl method (27), and the protein content was calculated using the conversion factor (6.25) of nitrogen-protein. Considering the protein content in the defatted cacao powder as 100%, the protein content in each cacao protein powder was calculated as the percentage yield.

### Optimized preparation procedure for cacao protein and lignin powders from defatted cacao powder

**FIGURE 1:**
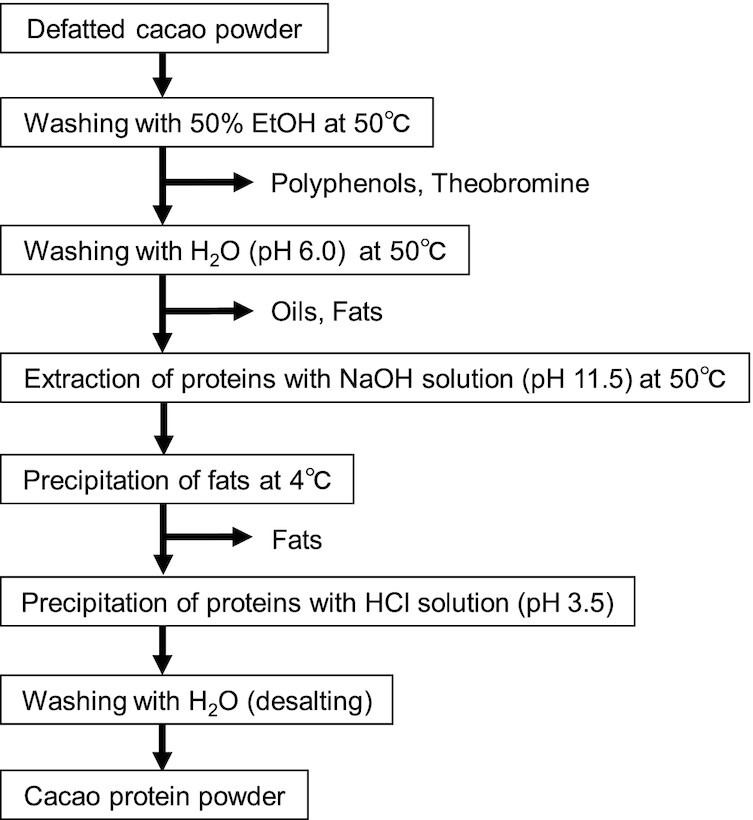
Extraction and purification procedure for cacao protein powder from defatted cacao powder.

The concrete preparation method of cacao protein powder established is as follows (**Figure 1**). One hundred twenty grams of the defatted cacao powder was agitated in 1.5 L of 50% EtOH for 60 min at 50°C. The agitated solution was centrifuged at 18,800 × *g* for 30 min at 25°C, and the supernatant containing polyphenols and theobromine was discarded (28, 29). This step was repeated once. Next, the residue was agitated in 1.8 L of water (pH 6.0) for 30 min at 50°C. The solution was then centrifuged at 18,800 × *g* for 30 min at 25°C, and the supernatant containing oils and fats was discarded. This step was repeated once. Any fats attached to the inner wall of the centrifuge tube were also removed. Cacao proteins were then extracted from the residue with 1.8 L of NaOH solution (pH 11.5) at 50°C for 60 min under agitation. The solution was then centrifuged at 18,800 × *g* for 30 min at 25°C, and the supernatant was incubated at 4°C for 16 h. After the incubation, the solution was centrifuged at 18,800 × *g* for 30 min at 4°C, and the supernatant removed from the centrifuge tube was again centrifuged at 36,000 × *g* for 20 min at 4°C. The supernatant was filtered through ADVANTEC No. 2 filter paper (Advantec Toyo Kaisha, Ltd.) to remove fats. The filtrate was adjusted to pH 3.5 with 6 M HCl to precipitate cacao proteins, then centrifuged at 18,800 × *g* for 30 min at 4°C. After removing the supernatant, the precipitant was agitated in 1.8 L of water for 20 min at 25°C. The agitated solution was centrifuged at 18,800 × *g* for 60 min at 4°C, and the supernatant containing salts and acids was discarded. This step was repeated once. The final precipitant was dried to obtain a cacao protein powder. This small-scale procedure to obtain the cacao protein powder was repeated. The amount of nitrogen in the cacao protein powder was measured by the Kjeldahl method (27), and the protein amount was calculated using the conversion factor (6.25) of nitrogen-protein. The cacao protein powder was dissolved in 0.2 M NaOH solution at 50°C, then the protein amount was also measured by the Bradford method with BSA as the standard (26).

Cacao lignin powder was prepared from the defatted cacao powder by the Southgate method (30). The amount of lignin in the cacao protein powder and cacao lignin powder was also measured by the Southgate method (30).

### Analysis of theobromine and caffeine by high performance liquid chromatography (HPLC)

Theobromine and caffeine were extracted and measured essentially as previously described (31, 32). The cacao protein powder was extracted with 70% EtOH. The extract solution was centrifuged at 20,000 × *g* for 30 min at 4°C and the supernatant was injected into the HPLC equipped with a TSKgel ODS-120T column (4.6 mm i.d. × 30 cm; Tosoh) eluted with a linear gradient of 0 to 25% EtOH in 1% acetic acid at 0.8 mL/min. The peaks corresponding to theobromine and caffeine were monitored with a UV detector at 280 nm.

### Digestibility of cacao proteins

Casein sodium was purchased from FUJIFILM Wako Pure Chemical (Osaka, Japan). A total of 4.3 g of the cacao protein powder or casein sodium was dissolved in water and adjusted to pH 2, then 43 mg of pepsin (1:10,000; FUJIFILM Wako Pure Chemical) was added to each protein solution and incubated at 37°C for 4 h. After incubation, each solution was adjusted to pH 10, then 86 mg of pancreatin (FUJIFILM Wako Pure Chemical) was added to each protein solution and incubated at 37°C for 16 h. After incubation, each pH was adjusted to 2, then the precipitated proteins were washed 5 times with water. The precipitant was collected by centrifugation at 20,400 × *g*for 30 min at 25°C, then dried. The nitrogen content of the undigested proteins was measured by the Kjeldahl method (27), and the protein amount was calculated using the conversion factor (6.25) of nitrogen-protein. Digestibility of cacao proteins was calculated by comparing the quantity of precipitated protein content, treated and nontreated with the digestive enzymes.

### Experimental design of animal study

All animal experiments were approved by the Welfare Committee of Laboratory Animals of Charles River Japan (Ibaraki, Japan) and conducted in accordance with its Guidelines for Care and Use. Six-week-old female BALB/c AnN CrlCrlj mice bred by Charles River Japan, Inc., were fed ad libitum in a room maintained at 23 ± 2°C with a 12-h light/dark cycle. After the mice were fed a solid control diet (AIN-93 G diet with 5.0% corn starch instead of 5.0% cellulose; **Table 1**) for 6 d, they were divided into 3 diet groups (*n *= 10 in each group) so as to have the same average feed intake and body weight, then the mice in each group were pair-fed ad libitum for 8 d a solid control diet, a solid cacao lignin diet (AIN-93 G diet with 1.586% cacao lignin powder and 3.414% corn starch instead of 5.0% cellulose; Table 1), or a solid cacao protein and lignin diet (AIN-93 G diet with 3.5% cacao protein powder and 1.5% corn starch instead of 5.0% cellulose; Table 1). The location of cages with individually housed mice was randomized in the room. The feces were collected as 2 bulked samples from days 1 to 4 and days 5 to 8 on each diet from individually housed mice. The collected feces were freeze-dried and weighed.

**FIGURE 3:**
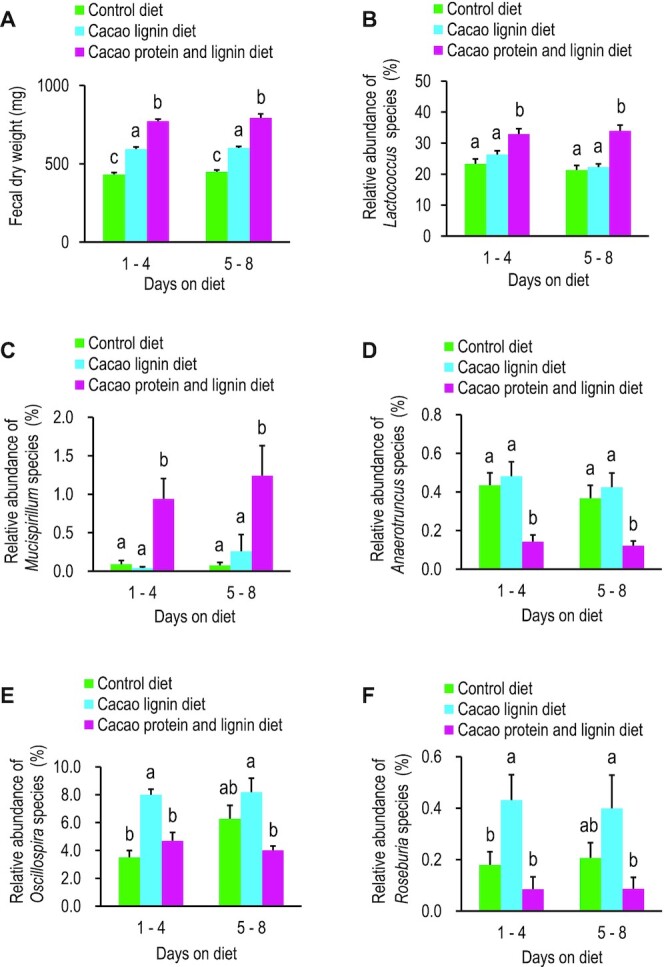
Fecal dry weight and relative abundance of 5 bacterial species in mice that ingested cacao protein and lignin, cacao lignin, or control diets. (A) Fecal dry weight of mice that ingested cacao protein and lignin, cacao lignin, or control diets. Feces were collected as 2 bulked samples from days 1 to 4 and days 5 to 8 on each diet from individually housed mice. Values are means ± SEMs (*n* = 10). Different letters indicate significant differences between diet groups by 1-factor ANOVA with Tukey's test (*P *< 0.05). Relative abundance of *Lactococcus* (B), *Mucispirillum* (C), *Anaerotruncus* (D), *Oscillospira* (E), and *Roseburia* (F) species in mice that ingested cacao protein and lignin, cacao lignin, or control diets. Values are means ± SEMs (*n* = 10). Different letters indicate significant differences between diet groups for a genus by 1-factor ANOVA with Tukey's test (*P *< 0.05).

**TABLE 1:** Composition of diets used in the feeding experiment

		g/100 g diet	
Component	Control diet	Cacao lignin diet	Cacao protein and lignin diet
Cacao protein powder	0.00	0.00	3.50
Cacao lignin powder	0.00	1.586	0.00
Corn starch	44.7486	43.1626	41.2486
α-Corn starch	13.2	13.2	13.2
Sucrose	10.0	10.0	10.0
Casein	20.0	20.0	20.0
l-Cystine	0.3	0.3	0.3
Soybean oil	7.0	7.0	7.0
AIN-93 G mineral mixture	3.5	3.5	3.5
AIN-93 vitamin mixture	1.0	1.0	1.0
Choline bitartrate	0.25	0.25	0.25
*t*-Butylhydroquinone	0.0014	0.0014	0.0014
Cacao lignin content, g	0.00	1.22	1.22
Cacao protein content, g	0.00	0.00	1.97
Total protein, g	20.30	20.30	22.27
Energy, kcal	419.99	413.65^1^	405.99^1^

1 Energy of cacao protein powder and cacao lignin powder was calculated as 0 kcal.

### Next-generation sequencing–based 16S rRNA microbial profiling

Bacterial DNA was extracted from the mouse feces using a QIAamp Fast DNA Stool Mini Kit (Qiagen). The V3–V4 region of 16S rRNA was amplified using region-specific primers (forward: 341F; reverse: 805R) and KAPA HiFi HotStart ReadyMix (KAPA Biosystems) (33). The index PCR was performed using Nextera XT Index Kit v2 Set A (Illumina), and the PCR amplicon was purified using Agencourt AMPure XP beads (Beckman Coulter). The DNA libraries were quantified using a Qubit Assay Kit (ThermoFisher Scientific) and were sequenced on a MiSeq System (Illumina).

The sequence data were analyzed using Quantitative Insights Into Microbial Ecology (QIIME), version 1.8.1, software (34). Sequences were removed when the quality score fell below 20 as determined using PRINSEQ software (35). After filtering, a total of 8,336,989 reads were obtained from 6 samples, with an average of 138,950 reads per sample. The sequences were normalized by rarefaction using QIIME. The remaining samples were grouped into operational taxonomic units (OTUs) at a minimum of 97% similarity by using QIIME's UCLUST method based on the Greengenes database.

### Statistical analysis

All data are expressed as means ± SEMs, and *P* < 0.05 was considered statistically significant. Differences in digestibility between each protein were determined using Student's *t* test. Differences in body weight, food intake, fecal dry weight, and relative abundance of intestinal bacteria between each diet group were determined using 1-factor ANOVA with Tukey's test.

## Results

### Comparison of protein amounts extracted from various seeds with a neutral aqueous solution

**FIGURE 2:**
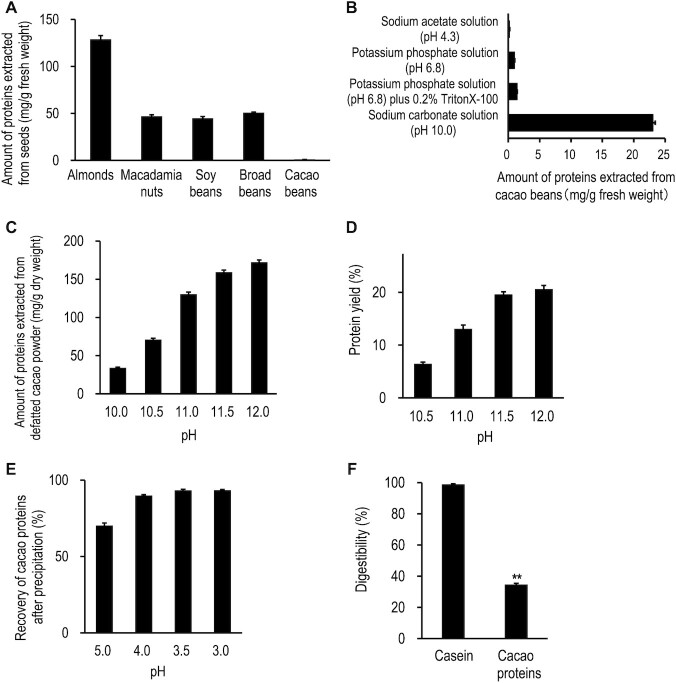
Search for a suitable extraction and purification method of proteins from cacao beans and digestibility of cacao proteins. (A) Amount of proteins extracted with a neutral aqueous solution (40 mM potassium phosphate solution, pH 6.8) from various seeds (mean ± SEM, *n* = 3). (B) Amount of proteins extracted with various solutions from cacao beans (mean ± SEM, *n* = 3). (C) Amount of proteins extracted from defatted cacao powder under various alkaline conditions (pH 10.0–12.0) (mean ± SEM, *n*  = 3). (D) Protein yield of cacao protein powder prepared from defatted cacao powder under various alkaline extraction conditions (pH 10.5–12.0). Considering the protein content in the defatted cacao powder as 100%, the protein content in each cacao protein powder was calculated as the percentage yield (mean ± SEM, *n* = 3). (E) Recovery of cacao proteins after precipitation at various pHs (pH 3.0–5.0). The total amount of cacao proteins before precipitation was taken as 100%; the amount of cacao proteins precipitated at each pH was calculated as the percentage of total recovered (mean ± SEM, *n* = 3). (F) Digestibility of cacao proteins and casein. The digestibility of cacao proteins was compared with that of casein using Student's *t* test (mean ± SEM, *n* = 3). **P < 0.01.

In general, plant proteins can be extracted with neutral aqueous solutions. For example, soy proteins are extracted with sodium phosphate buffer (pH 7.4) (36) and have been reported to have several beneficial effects on human health such as cardiovascular disease prevention (37). Therefore, we examined whether cacao proteins can be also extracted with the neutral aqueous solution. As shown in **Figure 2**A, high amounts of proteins were extracted from almonds, macadamia nuts, soybeans, and broad beans with a potassium phosphate solution (pH 6.8), but very low amounts of proteins (1.01 ± 0.02 mg/g) were extracted from cacao beans. Thus, a neutral aqueous solution is not suitable for extracting cacao proteins.

### Search for a suitable extraction and purification method of proteins from cacao beans

In general, membrane proteins can be extracted by solutions containing non-ionic surfactants such as Triton X-100 and Tween 20. So, we examined the effect of non-ionic surfactant on the extraction of cacao proteins. However, the addition of 0.2% Triton X-100 had no effect on the extraction (Figure 2B).

When we examined the effect of pH on the extraction, low amounts of protein (0.220 ± 0.009 mg/g) were extracted at pH 4.3, whereas high amounts of proteins (23.1 ± 0.4 mg/g) were extracted at pH 10.0, approximately 23 times more than those at pH 6.8 (1.01 ± 0.02 mg/g) (Figure 2B), suggesting that cacao proteins were effectively extracted under alkaline conditions. This result is consistent with our previous finding that cacao proteins were efficiently extracted with alkaline solutions (38). The tests of various alkaline conditions showed that the higher the pH, the higher the protein amount extracted (Figure 2C). The highest yield of cacao proteins was obtained with pH 11.5 and 12.0 (Figure 2D), so we used pH 11.5 for the extractions. Since approximately 93% or more of the cacao proteins extracted with the alkaline aqueous solution were precipitated below pH 3.5 (Figure 2E), we used pH 3.5 to precipitate the proteins.

From these results, the optimized preparation procedure for cacao protein powder was established as described in the Methods section (Figure 1). From 2,400g of the defatted cacao powder, 203 g of cacao protein powder was obtained. The content percentages of protein and lignin contained in the cacao protein powder were 56.4% (Kjeldahl method) and 34.9%, respectively. Also, the content percentage of protein in the cacao protein powder was 63.0% (Bradford method). The protein yield after these extraction and purification steps was 19.6% (Kjeldahl method). This preparation method gave the highest protein yield among the various preparation conditions, but it did not exclude lignin.

Cacao lignin powder was prepared from the defatted cacao powder by the Southgate method. From 700 g of the defatted cacao powder, 145 g of the cacao lignin powder was obtained. The content percentage of lignin contained in the cacao lignin powder was 77.0%.

### Digestibility of cacao proteins

Because the cacao proteins are alkaline-soluble, we hypothesized that the proteins might be modified by components such as saccharides. If so, the modifications might render the proteins resistant to proteases. To test our hypothesis, we compared the digestibility of cacao proteins with that of casein using pepsin and pancreatin. The data presented in Figure 2F show that the digestibility of cacao proteins was 34.6 ± 0.7%, clearly lower than that of casein (98.9 ± 0.4%). This result indicates that cacao proteins are resistant to digestive enzymes.

We performed the digestion experiment with pancreatin at pH 10 to dissolve the cacao proteins, but the intestinal pH in mouse is below 5.2 (39). When the digestion experiment with pancreatin is performed at pH 5.2, it is expected that the cacao proteins will precipitate, resulting in a further decrease in the digestibility of the precipitated cacao proteins.

### Effect of ingestion of cacao proteins on defecation in mice

Since cacao proteins were difficult to be digested by digestive enzymes such as pepsin and pancreatin (Figure 2F), we speculated that they would pass into the colon and be metabolized in the same manner as oligosaccharides by intestinal bacteria (11–13) and thus lead to an increase in defecation (5, 17). To test this hypothesis, we examined whether the ingestion of cacao proteins promotes defecation in mice.

Although our extraction and purification method yielded the greatest amount of proteins among the various trial preparations, it did not exclude lignin. Therefore, to reveal the effect by cacao proteins alone, we compared the fecal weight of mice fed the cacao protein and lignin diet with that of mice fed the same amount of the cacao lignin diet (Table 1). As shown in **Table 2**, the body weight and food intake did not differ significantly between mice fed the cacao protein and lignin diet and the cacao lignin diet during the test days (*P* > 0.05). The fecal dry weights of mice that ingested each diet are shown in **Figure 3**A. The total fecal dry weight of mice fed the cacao protein and lignin diet on days 1 to 4 and days 5 to 8 was significantly greater than that of mice fed the cacao lignin diet on days 1 to 4 and days 5 to 8, respectively. Thus, the ingestion of cacao proteins clearly promoted defecation in the mice.

**TABLE 2:** Food intake and body weight for mice in each diet in feeding experiment^1^

	Control diet	Cacao lignin diet	Cacao protein and lignin diet
Food intake for 8 d, g/mouse	27.7 ± 0.4	27.3 ± 0.4	27.8 ± 0.6
Body weight, g/mouse			
Initial	20.4 ± 0.3	20.1 ± 0.2	20.3 ± 0.2
Final	19.8 ± 0.4	19.1 ± 0.3	19.3 ± 0.6

1 Values are means ± SEM, *n *= 10.

### Effect of ingestion of cacao proteins on mouse intestinal microbiota

Because the cacao proteins promoted defecation in mice, they might also alter the intestinal microbiota. In the next-generation sequencing (NGS)-based 16S rRNA microbial profiling of the bacteria in the fecal samples, 129 bacterial species were detected (**Table 3**). To find bacteria reliably changed in the population by ingestion of cacao proteins, we selected the species significantly changed in relative abundance on both days 1 to 4 and days 5 to 8 (Table 3).

**TABLE 3:** Relative abundance of bacterial species in mice that ingested cacao protein and lignin, cacao lignin, or control diets^1^

	Relative abundance, %					
Bacterial species	1–4 Days fed the diet			5–8 Days fed the diet		
	Control diet	Cacao lignin diet	Cacao protein and lignin diet	Control diet	Cacao lignin diet	Cacao protein and lignin diet
*Adlercreutzia*	0.44 ± 0.08	0.62 ± 0.10	0.59 ± 0.09	0.38 ± 0.06	0.52 ± 0.05	0.41 ± 0.10
*Akkermansia*	27.29 ± 2.27^a^	3.49 ± 1.90^b^	5.97 ± 2.54^b^	18.48 ± 2.84^a^	3.23 ± 2.32^b^	0.55 ± 0.53^b^
*Alistipes*	0.01 ± 0.00	0.03 ± 0.02	0.03 ± 0.01	0.03 ± 0.01	0.02 ± 0.01	0.03 ± 0.01
*Allobaculum*	0.00 ± 0.00	0.18 ± 0.17	0.00 ± 0.00	0.01 ± 0.01	0.00 ± 0.00	0.00 ± 0.00
*Anaeroplasma*	0.00 ± 0.00	0.10 ± 0.05	0.00 ± 0.00	0.00 ± 0.00	0.12 ± 0.06	0.00 ± 0.00
Anaerotruncus*	0.44 ± 0.06^a^	0.48 ± 0.07^a^	0.14 ± 0.04^b^	0.37 ± 0.07^a^	0.42 ± 0.07^a^	0.12 ± 0.02^b^
*Bacteroides*	10.78 ± 3.25	7.06 ± 2.19	4.07 ± 1.34	14.67 ± 3.58	10.36 ± 2.65	6.12 ± 2.11
*Bilophila*	0.06 ± 0.06	0.00 ± 0.00	0.10 ± 0.10	0.16 ± 0.16	0.00 ± 0.00	0.14 ± 0.09
*Butyricicoccus*	0.01 ± 0.00	0.01 ± 0.01	0.05 ± 0.03	0.03 ± 0.01	0.02 ± 0.01	0.06 ± 0.03
*Coprobacillus*	0.06 ± 0.01^a^	0.00 ± 0.00^b^	0.00 ± 0.00^b^	0.05 ± 0.01^a^	0.00 ± 0.00^b^	0.00 ± 0.00^b^
*Coprococcus*	0.40 ± 0.70	0.86 ± 0.28	0.28 ± 0.05	0.54 ± 0.18	0.62 ± 0.09	0.21 ± 0.02
*Corynebacterium*	0.00 ± 0.00	0.00 ± 0.00	0.02 ± 0.02	0.01 ± 0.01	0.01 ± 0.01	0.00 ± 0.00
*Dehalobacterium*	0.09 ± 0.02^a,b^	0.11 ± 0.02^a^	0.05 ± 0.01^b^	0.10 ± 0.02	0.10 ± 0.02	0.06 ± 0.01
*Dorea*	1.87 ± 0.14^a^	0.57 ± 0.10^b^	0.22 ± 0.07^b^	1.46 ± 0.18^a^	0.49 ± 0.08^b^	0.17 ± 0.03^b^
*Escherichia*	0.09 ± 0.04	0.04 ± 0.01	0.04 ± 0.02	0.14 ± 0.05	0.26 ± 0.12	0.23 ± 0.21
*Lactobacillus*	2.59 ± 0.66^b^	8.02 ± 1.90^a^	3.66 ± 0.38^b^	2.65 ± 0.85	8.41 ± 3.20	4.11 ± 1.56
Lactococcus*	23.32 ± 1.56^a^	26.34 ± 1.16^a^	32.90 ± 1.74^b^	21.32 ± 1.44^a^	22.22 ± 1.06^a^	33.97 ± 1.84^b^
Mucispirillum*	0.09 ± 0.05^a^	0.04 ± 0.02^a^	0.94 ± 0.26^b^	0.07 ± 0.04^a^	0.26 ± 0.22^a^	1.24 ± 0.39^b^
Oscillospira*	3.52 ± 0.45^b^	7.99 ± 0.41^a^	4.69 ± 0.59^b^	6.27 ± 0.97^a,b^	8.18 ± 1.01^a^	4.00 ± 0.32^b^
*Parabacteroides*	6.70 ± 0.75	4.82 ± 0.88	7.95 ± 1.44	7.36 ± 1.15	5.19 ± 0.92	8.45 ± 1.95
Roseburia*	0.17 ± 0.05^b^	0.43 ± 0.10^a^	0.09 ± 0.05^b^	0.21 ± 0.06^a,b^	0.40 ± 0.13^a^	0.09 ± 0.04^b^
*Ruminococcus*	1.58 ± 0.56	1.38 ± 0.50	2.92 ± 1.41	1.27 ± 0.42	0.82 ± 0.13	1.82 ± 0.80
*Shuttleworthia*	0.03 ± 0.01	0.02 ± 0.01	0.02 ± 0.00	0.04 ± 0.01^a^	0.01 ± 0.00^b^	0.02 ± 0.00^a,b^
*Staphylococcus*	0.01 ± 0.01	0.04 ± 0.02	0.00 ± 0.00	0.01 ± 0.00	0.05 ± 0.04	0.00 ± 0.00
*Streptococcus*	0.14 ± 0.04	0.27 ± 0.07	0.24 ± 0.08	0.17 ± 0.03	0.24 ± 0.06	0.26 ± 0.08

1 Values are means ± SEM. Bacterial species mainly identified in next-generation sequencing (NGS)–based 16S rRNA microbial profiling are listed in the table. Different letters indicate significant differences between diet groups by 1-factor ANOVA with Tukey's test (*P *< 0.05). *These bacteria represent species significantly changed in relative abundance on both days 1 to 4 and days 5 to 8 by the ingestion of cacao proteins.

The relative abundance of *Lactococcus* and *Mucispirillum* species in mice fed the cacao protein and lignin diet was significantly higher than in mice fed the cacao lignin diet, whereas there was no significant difference in their relative abundance between mice fed the cacao lignin diet and the control diet (Figure 3B, C). These results indicate that cacao proteins, but not cacao lignin, increased the relative abundance of *Lactococcus* and *Mucispirillum* species.

The relative abundance of *Anaerotruncus*, *Oscillospira*, and *Roseburia* species in mice fed the cacao protein and lignin diet was significantly lower than in mice fed the cacao lignin diet (Figure 3D–F). The relative abundance of *Anaerotruncus* species between mice fed the cacao lignin diet and the control diet did not differ significantly (Figure 3D), whereas that of *Oscillospira* and *Roseburia* species in mice fed the cacao lignin diet tended to be higher than in mice fed the control diet (Figure 3E, F). These results indicate that cacao proteins, but not cacao lignin, decreased the relative abundance of *Anaerotruncus*, *Oscillospira*, and *Roseburia* species.

## Discussion

Castillejo et al. (40) reported that the ingestion of cocoa husk supplements by children with idiopathic chronic constipation tended to increase the number of bowel movements. Similarly, Jenkins et al. (41) reported that fecal quantity and defecation frequency in healthy normolipidemic subjects who had ingested cocoa bran cereal (total dietary fiber: 25.0 g/d) were higher than in those who had ingested low-fiber cereal (total dietary fiber: 5.6 g/d). These studies show that cacao fibers containing lignin, cellulose, and hemicellulose promote defecation. However, the effects of cacao proteins on defecation were still unknown, because the proteins were difficult to extract and purify from cacao beans. In the present study, we established a new extraction and purification method for cacao proteins and found that the ingested indigestible cacao proteins not only promoted defecation but also altered the intestinal microbiota in mice. This study is the first to show that the ingestion of cacao proteins promoted defecation and altered the intestinal microbiota in animals.

First, we measured the amount of theobromine and caffeine, which are the main nonprotein nitrogenous compounds in cacao beans (42), in the cacao protein powder because the Kjeldahl method measures these nonprotein nitrogenous compounds as proteins. Since the cacao protein powder contained only 0.00103% theobromine and 0.00062% caffeine, we are confident that the 56.4% protein content measured by the Kjeldahl method is mostly composed of protein. Using the Bradford method to measure the protein content, we found that the 63.0% protein content in the cacao protein powder was close to the 56.4% protein content determined by the Kjeldahl method. Thus, this protein content of 56.4% should be accurate.

In addition to the 56.4% protein content, the cacao protein powder obtained using this new method contained 34.9% lignin. The other 8.7% is considered to be mainly sugar chains bound to proteins, because the 56.4% cacao protein content measured by the Kjeldahl method was calculated without consideration of any sugar-chain content. Hence, the cacao protein powder is mostly composed of protein and lignin, suggesting that no compounds other than cacao protein and lignin are involved in promoting defecation and altering the intestinal microbiota in mice.

On the basis of the alkaline-solubility and protease resistance of cacao proteins, we speculate that the proteins are modified by components such as saccharides during the fermentation or roasting of cacao beans. If cacao proteins react with saccharides in a Maillard reaction during the roasting, basic amino acids, such as lysine or arginine, would combine with the saccharides. As a result, cacao proteins that have only carboxyl groups might readily dissolve in an alkaline solution. Alternatively, the proteins might originally have been alkaline-soluble and protease-resistant before they were roasted. In any case, the cacao proteins need to be further characterized.

Among the 129 bacterial genera detected in the NGS-based 16S rRNA microbial profiling of bacterial communities in the fecal matter from mice fed the cacao protein diet (Table 3), *Lactococcus*, *Mucispirillum*, *Anaerotruncus*, *Oscillospira*, and *Roseburia* species mainly changed in relative abundance (Figure 3B–F). We paid most attention to the increase in *Lactococcus* species. In our animal experiments, the ingestion of cacao lignin as well as cacao proteins also promoted defecation in the mice (Figure 3A). However, the *Lactococcus* species increased after the mice ingested cacao proteins, but not after they ingested cacao lignin (Figure 3B), suggesting that cacao proteins might be preferentially recognized by *Lactococcus* species in intestinal microbiota. *Lactococcus* species were the most abundant bacteria among the mouse intestinal microbiota in our study, representing more than 20% of the total bacterial population (Table 3). Furthermore, as a probiotic, *Lactococcus lactis*, mainly used to ferment foods, plays important roles in promoting gut health by producing short-chain fatty acids, promoting defecation, and enhancing intestinal immune function in mice and humans (43–46). If cacao proteins are directly metabolized by *Lactococcus* species and they promote defecation in the same manner as oligosaccharides such as fructo-oligosaccharides and galacto-oligosaccharides (5, 11–13, 17), we could say that cacao proteins function as a new prebiotic in mice. *Mucispirillum* species also increased after the ingestion of cacao proteins, but not cacao lignin (Figure 3C), suggesting that cacao proteins might also be preferentially recognized by *Mucispirillum* species. However, the population of *Mucispirillum* species is very low (0.94–1.24%), so the metabolism of cacao proteins by this species is not likely to be responsible for promoting defecation in mice.

We also focused on the decrease in *Oscillospira* species in mice after the ingestion of cacao proteins (Figure 3E) because the relative abundance of *Oscillospira* species in constipated adults was found to be significantly higher than in nonconstipated adults in a Chinese gut microbiome project (47) and positively correlated with constipation, especially in a female population with chronic constipation (48). These studies show that *Oscillospira* species might contribute to constipation in humans. Thus, the ingestion of cacao proteins might promote defecation in mice, in part by decreasing the population of *Oscillospira* species.

*L. lactis* also has anti-inflammatory activity in the colon, which leads to subsequent prevention of IBD in an IBD model mouse (49, 50). *Mucispirillum schaedleri* is reported to compete for anaerobic respiration substrates with *Salmonella enterica* serovar Typhimurium and to interfere with virulence factor expression, thereby protecting against *Salmonella* colitis in mice (51). Thus, the intake of cacao proteins might also help prevent IBD or protect against *Salmonella* colitis in addition to promoting defecation in mice.

When the feces of healthy adults were added to batch fermentations using proteins and peptones as the sole fermentable substrates, the number of *Anaerotruncus* species was highly correlated with the production of ammonium, indole, and *p*-cresol (52), known as humic substances in the gut. Thus, the decrease in the population of *Anaerotruncus* species after the ingestion of cacao proteins might also lead to a decrease in the accumulation of humic substances. We expect the intake of cacao proteins to provide various health benefits, such as diminishing IBD, protecting against *Salmonella* colitis, and decreasing the level of humic substances, as well as promoting defecation. If we are to elucidate the full health benefits of cacao proteins, further studies using mice for IBD or *Salmonella* colitis and analysis of humic substances are now needed.

Since dark chocolate contains high amounts of cacao proteins as well as polyphenols, the consumption of dark chocolate might alleviate constipation in human and provide other benefits. Thus, further studies on the effects of dark chocolate on defecation, intestinal microbiota, and the gastrointestinal tract in humans are essential. If the ingestion of dark chocolate alleviates constipation and alters the intestinal microbiota, perhaps cacao proteins will be recognized as a functional ingredient, in addition to cacao polyphenols, in chocolate.

## Data Availability

Data described in the manuscript are available upon pending request.

## Notes

Supported by Teikyo University, Japan, to revitalize educational activities.

Author disclosures: The authors report no conflicts of interest.

This article is dedicated to the memory of Mr Takumi Sugamata, who passed away in April 2022.

Abbreviations used: BSA, bovine serum albumin; IBD, inflammatory bowel disease; NGS, next-generation sequencing; QIIME, quantitative insights into microbial ecology.
